# Supplementary material for: Male coloration affects female gestation period and timing of fertilization in the guppy (Poecilia reticulata)
Source: PLoS One. 2021 Dec 2;16(12):e0261004. doi: 10.1371/journal.pone.0261004 (PMC8639057; doi:10.1371/journal.pone.0261004)
Supplement: Attachment — Submitted filename: PONE-D-21-14329_EDITED.pdf [file pone.0261004.s002.pdf]

PLOS ONE

Male coloration affects female gestation period and timing of fertilization in the guppy (<i>Poecilia reticulata<i>)

--Manuscript Draft--

**Manuscript Number:**	PONE-D-21-14329
**Article Type:**	Research Article
**Full Title:**	Male coloration affects female gestation period and timing of fertilization in the guppy (<i>Poecilia reticulata<i>)
**Short Title:**	Male guppy coloration affects female gestation period and fertilization timing
**Corresponding Author:**	Aya Sato<br>Gunma University: Gunma Daigaku<br>Maebashi, Gunma JAPAN
**Keywords:**	Sexual selection; Cryptic female choice; Reproductive strategy; Male coloration; Guppy
**Abstract:**	Trade-up hypothesis is a female behavioral strategy related to multiple mating. In this hypothesis, females could produce high quality offspring while avoiding the risk of losing reproductive opportunities, through they non-selectively mate with males at first mating and re-mate with more attractive males than previously mated males. As an internal mechanism to realize this behavioral strategy, we predicted that females immediately fertilized their eggs when they mated with attractive males, whereas, females delayed fertilization when they mated with unattractive males in order to trade-up sires of offspring. The guppy (<i> Poecilia reticulata <i>) is an ovoviviparous fish with internal fertilization, and females show a clear mate preference based on the area of male orange coloration on their body. In addition, it is known that females show a re-mating strategy following the trade-up hypothesis. We tested whether the attractiveness of mated males affected to the gestation period and timing of fertilization. Females were paired with either colorful males or drab males, and days from mating to parturition were compared. In addition, we dissected the abdomen of the females at intervals of several days after mating and observed whether the eggs were fertilized. As a result, the gestation period (the number of days from pairing to parturition) in females that have paired with attractive colorful males was significantly shorter than that in females that paired with drab males. And, the females that have mated with colorful males had their eggs fertilized earlier compared with those that have mated with drab males. This finding shows that there is a female cryptic choice in guppies as they can control the timing of fertilization according to male attractiveness and indicates the possibility that the cryptic process affects female remating strategy.
**Order of Authors:**	Aya Sato
	Ryu-ichi Aihara
	Kenji Karino
**Additional Information:**	
**Question**	**Response**
**Financial Disclosure**<br><br>Enter a financial disclosure statement that describes the sources of funding for the work included in this submission. Review the submission guidelines for detailed requirements. View published research articles from *PLOS ONE* for specific examples.<br><br>This statement is required for submission	The authors received no specific funding for this work.

Powered by Editorial Manager® and ProduXion Manager® from Aries Systems Corporation

and **will appear in the published article** if the submission is accepted. Please make sure it is accurate.<br><br>**Unfunded studies**<br>Enter: *The author(s) received no specific funding for this work.*<br><br>**Funded studies**<br>Enter a statement with the following details:<br>• Initials of the authors who received each award<br>• Grant numbers awarded to each author<br>• The full name of each funder<br>• URL of each funder website<br>• Did the sponsors or funders play any role in the study design, data collection and analysis, decision to publish, or preparation of the manuscript?<br>• **NO** - Include this sentence at the end of your statement: *The funders had no role in study design, data collection and analysis, decision to publish, or preparation of the manuscript.*<br>• **YES** - Specify the role(s) played.<br><br>* typeset	
**Competing Interests**<br><br>Use the instructions below to enter a competing interest statement for this submission. On behalf of all authors, disclose any competing interests that could be perceived to bias this work—acknowledging all financial support and any other relevant financial or non-financial competing interests.<br><br>This statement is required for submission and **will appear in the published article** if the submission is accepted. Please make sure it is accurate and that any funding sources listed in your Funding Information later in the submission form are also declared in your Financial Disclosure statement.<br><br>View published research articles from *PLOS ONE* for specific examples.	The authors have declared that no competing interests exist.

Powered by Editorial Manager® and ProduXion Manager® from Aries Systems Corporation

**NO authors have competing interests**<br><br>Enter: *The authors have declared that no competing interests exist.*<br><br>**Authors with competing interests**<br><br>Enter competing interest details beginning with this statement:<br><br>*I have read the journal's policy and the authors of this manuscript have the following competing interests: [insert competing interests here]*<br><br>* typeset	
**Ethics Statement**<br><br>Enter an ethics statement for this submission. This statement is required if the study involved:<br><br>• Human participants<br>• Human specimens or tissue<br>• Vertebrate animals or cephalopods<br>• Vertebrate embryos or tissues<br>• Field research<br><br>Write "N/A" if the submission does not require an ethics statement.<br><br>General guidance is provided below. Consult the submission guidelines for detailed instructions. **Make sure that all information entered here is included in the Methods section of the manuscript.**	Female laparotomy treatments were approved by the Animal Care and Experimentation Committee Gunma University, Japan (permit numbers: 20-040). We declare that all experiments in this study were conducted according to the guidelines of the committee.<br>After females were anaesthetized in 0.7 mL/L 2-phenoxyethanol solution, they were euthanized by over-anesthetization using 2-phenoxyethanol.

Powered by Editorial Manager® and ProduXion Manager® from Aries Systems Corporation

**Format for specific study types**<br><br>**Human Subject Research (involving human participants and/or tissue)**<br>• Give the name of the institutional review board or ethics committee that approved the study<br>• Include the approval number and/or a statement indicating approval of this research<br>• Indicate the form of consent obtained (written/oral) or the reason that consent was not obtained (e.g. the data were analyzed anonymously)<br><br>**Animal Research (involving vertebrate animals, embryos or tissues)**<br>• Provide the name of the Institutional Animal Care and Use Committee (IACUC) or other relevant ethics board that reviewed the study protocol, and indicate whether they approved this research or granted a formal waiver of ethical approval<br>• Include an approval number if one was obtained<br>• If the study involved *non-human primates*, add *additional details* about animal welfare and steps taken to ameliorate suffering<br>• If anesthesia, euthanasia, or any kind of animal sacrifice is part of the study, include briefly which substances and/or methods were applied<br><br>**Field Research**<br><br>Include the following details if this study involves the collection of plant, animal, or other materials from a natural setting:<br>• Field permit number<br>• Name of the institution or relevant body that granted permission	
**Data Availability**<br><br>Authors are required to make all data underlying the findings described fully available, without restriction, and from the time of publication. PLOS allows rare exceptions to address legal and ethical concerns. See the PLOS Data Policy and FAQ for detailed information.	Yes - all data are fully available without restriction

A Data Availability Statement describing where the data can be found is required at submission. Your answers to this question constitute the Data Availability Statement and **will be published in the article**, if accepted.<br><br>**Important:** Stating 'data available on request from the author' is not sufficient. If your data are only available upon request, select 'No' for the first question and explain your exceptional situation in the text box.<br><br>Do the authors confirm that all data underlying the findings described in their manuscript are fully available without restriction?	
**Describe where the data may be found in full sentences. If you are copying our sample text, replace any instances of XXX with the appropriate details.**<br><br>• If the data are **held or will be held in a public repository**, include URLs, accession numbers or DOIs. If this information will only be available after acceptance, indicate this by ticking the box below. For example: *All XXX files are available from the XXX database (accession number(s) XXX, XXX.*).<br>• If the data are all contained **within the manuscript and/or Supporting Information files**, enter the following: *All relevant data are within the manuscript and its Supporting Information files.*<br>• If neither of these applies but you are able to provide **details of access elsewhere**, with or without limitations, please do so. For example:<br><br>*Data cannot be shared publicly because of [XXX]. Data are available from the XXX Institutional Data Access / Ethics Committee (contact via XXX) for researchers who meet the criteria for access to confidential data.*<br><br>*The data underlying the results presented in the study are available from (include the name of the third party*	All relevant data are within the manuscript and its Supporting Information files.

and contact information or URL).<br>• This text is appropriate if the data are owned by a third party and authors do not have permission to share the data.<br><br>* typeset	
Additional data availability information:	

Powered by Editorial Manager® and ProduXion Manager® from Aries Systems Corporation

Manuscript Click here to access/download;Manuscript;Manuscript.docx

Full title: Male coloration affects female gestation period and timing of fertilization in the guppy (*Poecilia reticulata*)

Short title: Male guppy coloration affects female gestation period and fertilization timing

Aya Sato[1*], Ryu-ichi Aihara[2, #a], Kenji Karino[2]

[1] Department of Science Education, Cooperative Faculty of Education, Gunma University, Maebashi, Japan

[2] Department of Biology, Tokyo Gakugei University, Tokyo, Japan

[#a] Current Address: Sunagawa High School, Tokyo, Japan

*Corresponding author

E-mail: satoaya@gunma-u.ac.jp (AS)

Abstract

Trade-up hypothesis is a female behavioral strategy related to multiple mating. In this hypothesis, females could produce high quality offspring while avoiding the risk of losing reproductive opportunities, through they non-selectively mate with males at first mating and re-mate with more attractive males than previously mated males. As an internal mechanism to realize this behavioral strategy, we predicted that females immediately fertilized their eggs when they mated with attractive males, whereas, females delayed fertilization when they mated with unattractive males in order to trade-up sires of offspring. The guppy (*Poecilia reticulata*) is an ovoviviparous fish with internal fertilization, and females show a clear mate preference based on the area of male orange coloration on their body. In addition, it is known that females show a re-mating strategy following the trade-up hypothesis. We tested whether the attractiveness of mated males affected to the gestation period and timing of fertilization. Females were paired with either colorful males or drab males, and days from mating to parturition were compared. In addition, we dissected the abdomen of the females at intervals of several days after mating and observed whether the eggs were fertilized. As a result, the gestation period (the number of days from pairing to parturition) in females that have paired with attractive colorful males was

significantly shorter than that in females that paired with drab males. And, the females that have mated with colorful males had their eggs fertilized earlier compared with those that have mated with drab males. This finding shows that there is a female cryptic choice in guppies as they can control the timing of fertilization according to male attractiveness and indicates the possibility that the cryptic process affects female remating strategy.

Introduction

All males are not equally successful in mating with females, and all males that succeeded in mating with female do not equally sire offspring when a female mates with multiple males. Male-male competition and female mate choice, which compose sexual selection, has the potential to continue post-copulation periods (following intromission or spawning) in the form of sperm competition and cryptic female choice [1,2]. Sperm competition is the post-copulatory equivalent of male-male competition and occurs when the ejaculates of two or more males compete for fertilization of a given set of ova [3]. Cryptic female choice corresponds to the post-copulatory female mate choice and occurs when females themselves bias sperm use in favor of particular males [4,5]. Therefore, males that succeed in sperm competition and/or that

are favored in cryptic female choice are able to sire more offspring among the multiple males that mated with a female. Eberhard [5] described a list of various female-controlled processes and activities during cryptic female choice, and they vary in ways that can modify the chance that a given copulation will result in offspring. To bias a male that sire offspring among multiple mated males female can control not only the transport, storage, and/or use of sperm from the mated males, but also mating or not with subsequent males [5].

Trade-up hypothesis is a female behavioral strategy involving multiple mating. When females choose mates at pre-copulation, they confront decisions to mate with a current male or to seek a subsequent male. If females would avoid mating with the current male to seek a more attractive male, it is possible that they could not find subsequent males and might miss the opportunity for reproduction. On the contrarily, if females would mate with the current male, they might miss the opportunity to find and mate with subsequent males with higher quality. The trade-up hypothesis describes a behavioral strategy in which females can maximize the genetic quality of their offspring when they encounter with multiple males sequentially [6,7]. The hypothesis suggests that in non-resource-based mating systems, with some degree of last-male sperm precedence, a female should mate with the first male that she encounters to

ensure fertilization of eggs, but subsequently mate preferentially with males of higher genetic quality. Sequential mate choice in female newts *Triturus vulgaris* [8] and crickets *Gryllus bimaculatus* [9] have supported this hypothesis. Thus, females are relatively indiscriminate at the first mating, but they become increasingly choosiness with each successive mating opportunity.

The guppy (*Poecilia reticulata*) is an ovoviviparous fish with internal fertilization and non-resource-based promiscuous mating system [10,11]. Female guppies show a clear mate preference in pre-copulation: females in many populations prefer males with large orange area on body [12-14]. Females mate with multiple males, and give birth to a brood with multiple paternity [15,16]. Pitcher et al. [17] proves that female guppies show a mating strategy that follows the trade-up hypothesis. They presented sequentially virgin females with two males of varying attractiveness (amount of orange coloration on their body). And they showed that female responsiveness to second males have were increased when the ornaments of second males have were more than the first males, compared to when the ornaments of second males have were less than the first males or were same as the first males. In addition, there was an overall tendency for last-male advantage in paternity, and this advantage was most exaggerated

when the second male was more ornamented than the first. Thus, female guppies may be able to maximize the genetic quality of their offspring through such a trade-up of mated males during successive matings. While Pitcher et al. [17] demonstrated the trade-up hypothesis in the guppy from the behavioral and paternity data, cryptic processes occurring in the female internally was unknown.

The cycle of egg production in guppies is non-superfetatious, where females give birth to a brood before the next ova are fertilized, and they carry a single brood at one time [11,18]. During development, embryos are nourished by the yolk that females deposits in the eggs prior to fertilization [10]. Fertilization in Poeciliids occurs over a period of one to five days after one to eight days after parturition [18]. The reproductive cycle of guppies typically lasts about 25–30 d, although there can be considerable variation [11]. For example, gestation period (days from mating to parturition) of virgin females vary approximately 22–65 d [19] and 20–60 d [20]. Gestation period is composed of two phases, the interval between insemination and fertilization (which may also include a yolk-loading phase) and the ensuing interval between fertilization and parturition [20]. Evans and Magurran [19] showed that gestation period in female guppies that paired with multiple males were shorter than those mated with a single male,

and females which paired with a single male might have delayed the process of fertilization for a more suitable mating. In addition, gestation period is shorter for female guppies that mated with colorful males than for those that mated with drab males [21]. Considering the above findings, we predict that, by mating with attracted males at the first time, females would immediately fertilize their ova, whereas females would delay the fertilization when they mated with unattractive males at the first time in order to trade-up males sired their offspring. In this study, we investigated whether the sexual attractiveness of mated male would affect gestation period and timing of fertilization in female guppies.

Materials and methods

Subject

Fish used in study were laboratory-reared descendants of feral guppies from the Hiji River (26º43´N, 128º11´E), Okinawa Island, southern Japan. Females in this population prefer males with larger orange area on body side than those with smaller ones [22,23]. Fish were reared in a laboratory at Gunma University. Water temperature was maintained at 27 ± 2 °C. Illumination was provided by fluorescent tubes, and light/dark regime was set at 12/12 h. All fish were fed

once daily with newly hatched brine shrimp and commercial flake food (Tetramin; Tetra Werke, Germany).

Males were reared in 40 L mixed-sex aquarium (45 × 30 × 30 cm) containing approximately 50 males and 50 females. To eliminate the influence of previous mating, we used virgin females that were reared in 12 L single-sex aquarium (37 × 22 × 25 cm) from 30–60 d after birth.

Experimental design

First, we compared gestation period (duration from mating to parturition) between females that mated with colorful males and those that mated with drab males. In addition, to examine the effect of male coloration on timing of fertilization, we observed whether the ova were fertilized or not by opening their abdomens between 2 to 22 d after pairing with colorful or drab males.

Ethics statement

Female laparotomy treatments were approved by the Animal Care and Experimentation Committee Gunma University, Japan (permit numbers: 20-040). We declare that all experiments

in this study were conducted according to the guidelines of the committee.

Test fish

We used matured individuals more than 5 months old as test fish. Before the start of the experiment, test fish were anesthetized with 2-phenoxyethanol solution, and body size (i.e. total and standard length) were recorded to the nearest 0.1 mm by using a vernier caliper.

In order to quantify body coloration, males were photographed on the left and right sides following the body size measurements. For photography, a digital camera (TG-3; Olympus, Tokyo, Japan) was placed at a distance of 4 cm above the male in petri dish containing 2-phenoxyethanol solution for anesthetization, and two lights (25 W) illuminated both sides of the petri dish from a position 20 cm away from fish. The recorded images were analyzed using Adobe Photoshop Elements 14 (Adobe Systems, San Jose, CA). The relative area of the orange spots was calculated as the ratio of the total area of orange spots to the total area of the body and caudal fin. We averaged the relative area of the orange spots from the right and left sides, and considered it as orange spot area of the male.

For pairing, a tetrad was created with two virgin females and two males. Two

females in a tetrad had approximately the same body sizes (within 2 mm difference in total length). Two males in a tetrad had approximately the same body sizes (within 2 mm difference in total length), while orange spot areas differed. We assigned individuals with a relatively larger orange spot area as colorful males and individuals with a relatively smaller orange spot area as drab males. Before pairing, the test females were placed in a small transparent tank (11 × 11 × 9 cm) that was subsequently placed in the male rearing aquarium (containing approximately 50 adult males) for 24 hours, in order to enhance mate choosiness of the female by contacting visually with various males.

Pairing

The pairing tank was a transparent plastic aquarium (18 × 10 × 11 cm) and was divided into two compartments (9 × 10 × 11 cm) using a transparent divider. The tank was filled with water having a depth of 8 cm and the bottom was covered with gravel. The water temperature and light condition was the same as mentioned previously. One tetrad was placed in the pairing tank. For an acclimation period, two males were placed in one compartment of a tank and two females were placed in the other compartment of the same tank. After a 24 h acclimation period,

one male and one female in each compartment were replaced, then one compartment contained a colorful male and a female and the other compartment contained a drab male and a female. During the mating period, pair of male and female freely mated for 24 h.

After pairing, the test males were individually housed for use again as a different tetrad, or removed to a stock aquarium. The females after paring were individually reared in the parturition tanks (10 × 15 × 9 cm) containing 1.2 L water and gravel. The females were allocated either parturition treatments or laparotomy treatments, then two females in same tetrad were allocated to the same treatment. As a shelter for the offspring, a fake aquatic plant made of thin green polyethylene tape were placed in the tanks containing females in the parturition treatments. These tanks were observed daily, and checked for newborn brood. When the females gave birth, we calculated the number of days from pairing to parturition.

Female laparotomies and discrimination of fertilization

The abdomens of females in the laparotomy treatment were opened between 2 and 22 d after pairing and the ova or embryos were extracted. We set a maximum of 22 d after pairing because the shortest gestation period was 25 d in the above parturition treatment. Two females in the

same tetrad were laparotomized on the same day. When the same pair of colorful and drab males were used for different tetrads, the females of different tetrads were laparotomized at different days after pairing. In addition, we also laparotomized nine virgin females as control treatment.

After females were anaesthetized in 0.7 mL/L 2-phenoxyethanol solution, they were euthanized by over-anesthetization using 2-phenoxyethanol. Using scissors and fine forceps, the ventral side of the abdomen was dissected starting from the cloaca to the anterior. The ovaries were extracted under a dissecting microscope and placed in a petri dish containing fresh water. The thin connective tissue of the ovary was carefully incised to isolate individual ova and embryos using fine tweezers. The ova and embryos were examined under a dissecting microscope to determine its developmental stage.

The determination of ova and embryonic developmental stage was referred from Haynes [24] and Martyn et al. [25]. When females held early-yolked ovum (Fig.1a) or mature ovum which fully yolked (Fig.1b), we considered their ovum to be unfertilized. When females had a blastodisc embryo (Fig.1c), primitive streak embryo (Fig.1d) or embryos after these stages (Fig.1e), we considered their ovum to be fertilized. If females had only small white immature

ova (Fig.1f), we excluded them from the analyses as these ova considered not ready for fertilization.

Fig. 1. Unfertilized ovum and fertilized ovum. a) early yolked ovum, b) mature ovum, c) blastodisc embryos, d) primitive streak embryo, e) developing embryo, and f) immature ova. Scale bar = 1 mm.

Statistical analyses

General estimating equations (GEE) was used to compare gestation period between females paired with colorful males and those paired with drab males. GEE analysis extends generalized linear models (GLMs) to account for repeated measures as well as allowing for linear and nonlinear models [26]. Tetrad identities were incorporated as repeated measures variable. We used the gestation period as a dependent variable; the male coloration (colorful / drab) as a fixed factor; brood size and female standard length as a covariate. The data was fitted to a normal distribution with an identity link function.

In order to examine whether the male coloration that females paired with affected the

timing of fertilization, we conducted GEE using binomial data. Values 0 and 1 were assigned to females that had unfertilized and fertilized ova, respectively; the 1/0 data were considered as a dependent variable. We used the male coloration (colorful / drab) as a fixed factor; the number of days from pairing as a covariate. Tetrad and male identities were entered in models as repeated measure variables, because some males were used in several different tetrads. The data were fitted to a binomial distribution with a logit link function. In addition, it was estimated that the inflection points of fertilization occurred in 50 % of females that were paired with either colorful or drab males. We performed logistic regression analyses and calculated the inflection points from the regression equation as $Y = 1/\exp[-(\alpha + \beta X)]$. All statistical tests were performed using SPSS version 26.0.0.0 and R version 3.3.3.

Results

We created 60 pairs used 54 colorful males and 51 drab males. And 79 tetrads were created from 60 male pairs and 158 females. The body size of colorful males was 16.4 ± 1.2 mm (standard length; mean ± standard deviation) and that of drab males was 16.4 ± 1.2 mm (paired t-test; n = 60 pair, t = 0.312, p = 0.714). The body size of females that paired with colorful

males was 21.8 ± 1.9 mm and the body size of females that paired with drab males was 21.8 ± 1.9 mm (paired t-test; n = 79 pair, t = -0.078, p = 0.938). The orange area of colorful males was 10.9 % ± 4.7 % and that of drab males was 2.9 % ± 1.2 % (paired t-test; n = 60 pair, t = 12.241, p < 0.001). We assigned the parturition treatment to females in 24 tetrads and the laparotomy treatment to females in 55 tetrads. All relevant data are within the manuscript and its Supporting Information files (S1).

In the parturition treatment, one female that paired with a colorful male and another that paired with a drab male did not give birth to a brood even 120 d after pairing. These data were excluded from analyses. The male colorations significantly affected gestation period (Table 1). The gestation period in females that paired with colorful males (mean ± standard deviation: 31.1 ± 7.3 d) was significantly shorter than that in females that paired with drab males (39.0 ± 9.8 d; Fig.2). Brood size was not affected by male coloration, although it was significantly affected by body size of females (Table 1).

Table 1. Result of GEE in parturition treatment

	B	95% Wald's confidence interval (lower)	95% Wald's confidence interval (upper)	Wald χ^2	*P*
Gestation period					
Male type (colorful / drab)	7.54	6.19	53.98	7.309	0.007
Brood size	0.15	-0.50	0.79	0.201	0.654
Female size	-0.04	-1.36	1.29	0.003	0.958

remove from model?

this is the graph we need to see

Brood size					
Male type (colorful / drab)	0.87	-2.77	4.52	0.221	0.639
Female size	1.39	0.51	2.27	9.513	0.002

Fig. 2 Female brood size and gestation period. Circles indicate females that paired with colorful males and cross indicate females that paired with drab males.

The virgin females had immature ova, early-yolked or mature ova (1 female; only immature ova; 2 females; only early-yolked ova; 4 females; only mature ova; 2 females; early-yolked and mature ova). In the laparotomy treatment, all 3 females that paired with a certain colorful male had an early-yolked ova when laparotomy was performed (8, 15, 22 d after pairing). Similarly, all 3 females that paired with a certain drab male had an early-yolked ova when laparotomy was performed (6, 12, 20 d after pairing). These data were excluded from the analyses because the males might have been infertile. In addition, four females (two of colorful pairs and two of drab pairs) were death before the laparotomy treatments. Eight females (four of colorful pairs and four of drab pairs) either had only small white immature ova or no ova at all. These data were also excluded from the analyses.

The number of days from pairing and male coloration (colorful / drab) significantly

affected the timing of fertilization (Table 2, Fig. 3). The inflection points where half the females had fertilized ova were 11.7 d for colorful pair and 18.1 d for drab pair.

Table 2. Result of GEE in laparotomy experiment

	B	95% Wald's confidence interval (lower)	95% Wald's confidence interval (upper)	Wald χ^2	*P*
Male type	-1.23	-2.31	-0.15	5.00	0.025
Days	0.24	0.15	0.33	27.51	<0.001

Fig. 3 Male coloration and timing of fertilization. Circles indicate females that paired with colorful males and crosses indicate females that paired with drab males. Solid and dotted curves indicate logistic regression lines of females that paired with colorful and drab males, respectively.

Discussion

We investigated whether the male orange spot area (indicating male sexual attractiveness) affect female gestation period and timing of fertilization. The gestation periods of females that mated with males possessing larger orange area was significantly shorter than that of female that mated with males possessing smaller orange area. The gestation period in the guppy is broadly comprising two phases: (1) the interval between insemination and fertilization (which may also

include a yolk-loading phase), and (2) the ensuing interval between fertilization and the parturition [20]. The timing of fertilization in females that paired with males possessing larger orange area was significantly earlier than those in females that paired with males possessing smaller orange area. Interestingly, the difference in mean gestation period between females that paired with colorful males and those that paired with drab males was 7.9 d, while the difference in days of inflection point that 50% of females were fertilizing their ova was 6.4 d, i.e., these differences were approximately the same. These results also suggest that difference in gestation periods caused by the difference in timing of fertilization according to mate ornamentation.

In the laparotomy treatments, at more than 18 d after pairing, some females had unfertilized ova when they had paired with drab males, whereas all females had fertilized ova when they had paired with colorful males. Therefore, it is possible that statistical analysis may have overestimated the effect of male coloration on the timing of fertilization because females that paired with drab males failed to fertilize their ova. For example, the drab males might fail to copulate or deliver sperm during copulation, or the sperm of drab males might fail to fertilize ova. Actually, it is known that male guppies with small orange area transfer less sperm to females than those with large orange area [27] (this is the result of female control [28]).

However, in the parturition treatment in this study, the fact that the number of females that did not give birth was the same regardless of mate coloration, rejects the possibility that the females that paired with drab males failed in mating or receiving sperm. In parturition treatment, the shortest gestation period was 25 d (this is regarded as the minimum time that was required for embryonic development to parturition from fertilization), whereas longest gestation period was 58 d. Therefore, it is possible that the ova are fertilized more than 18 d after pairing, because the gestation duration is 44 d (that is 25 d after fertilization) even if the ova are fertilized at 19 d after pairing.

We assumed that the difference in timing of fertilization depending on the male sexual ornamentation is a female cryptic choice that enables trade-up strategy in multiple mating. However, the difference in timing of fertilization can also be caused by the male. A positive relationship between carotenoid-based male ornamentation and sperm quality (velocity) is reported in several species [29-31]. In fact, it is known that colorful male guppies produce faster sperm [32,33] (but the results are contradictory [34,35]). Alternatively, male semen products, such as induction of ovulation or oviposition and resistance to further insemination by other males [36,37] might affect timing of fertilization. Effects on oviposition and resistance to

mating are particularly well-documented and widespread in insect and ticks [5], although it is not reported in the guppy. Such quality of sperm or semen products may affect to timing of fertilization. However, in the study used guppy, it is reported that the male orange area does not affect gestation period when females produce their brood under conditions excluding female control, considering male sexual attractiveness by the technique of artificial insemination [38,39]. This supports our assumption that the difference in timing of fertilization depending on the male sexual ornamentation is a cryptic effect of females.

The control of timing of fertilization (or oviposition after copulation) by females have been reported in several arthropods [40-43]. In these species, the lengthened period of female sexual receptivity due to delayed fertilization increases the opportunity for re-mating, as a result, females are able to bias fertilization towards preferred males [41]. By mating with colorful males that have large orange area, female guppies can gain indirect benefits that they produce offspring with high anti-predator ability [38] and algal-foraging ability [44] as well as sons with more attractive ornaments inherited form sire [45,46]. Virgin females are less choosy when they mate for the first time than in subsequent matings, and their probability of re-mating increases if the second male is more attractive than the first [17]. In addition, paternity is usually

biased towards the second male [17,47,48], possibly as a consequence of females biasing the number of sperm transferred during copulation to favor the most attractive male [49]. Consequently, female guppies are able to selectively produce higher quality offspring while avoiding the risk of losing reproductive opportunities. The result of the present study indicates that the control of fertilization timing by females is involved in such trade-up strategy.

Females that mate at first with unattractive males will gain the benefit of having offspring with high quality sired by the second attractive mates by delaying fertilization. However, if females do not encounter more attractive males despite delaying fertilization, they will lose future opportunities for reproduction. How long after first mating will the cost of delaying fertilization be higher than the benefits from trade-up? The duration of gestation in this study was 25–58 d. The duration of gestation in other studies were approximately 22–65 d [19] and 20–60 d [20]. Each maximum day is about twice the typical reproductive cycle (about 25–30 d [11]). Therefore, the loss of the opportunity of one reproductive cycle may be a large cost for females. The cost of delayed fertilization varies according to the expectation of remating with more attractive males or the remaining opportunity of reproduction. Further studies investigating the relationship between these factors and delayed fertilization will contribute to

understanding female trade-up strategy.

Acknowledgments

We thank Kaoru Yoshida for teaching the laparotomy technique. We are grateful to Aki Mieno, Ayaka Niwa, Mizuki Ogata and Yusuke Sakai for helping with animal care.

References

1. Birkhead TR, Møller AP. Sperm competition and sexual selection. London: Academic Press; 1998.
2. Birkhead TR, Pizzari T. 2002. Postcopulatory sexual selection. Nat Rev Genet.2002; 3:262–273.
3. Parker GA. Sperm competition and its evolutionary consequences in the insects. Biol Rev Cambr Philos Soc. 1970; 45:525–567.
4. Thornhill R. Cryptic female choice and its implications in the scorpionfly *Harpobittacus nigriceps*. Am Nat. 1983; 122:765–788.
5. Eberhard WG. 1996. Female control: sexual selection by cryptic female choice. Princeton,

NJ: Princeton University Press; 1996.

6. Halliday TR. The study of mate choice. In: Bateson P, editor. Mate choice. Cambridge: Cambridge University Press; 1983. p. 3–32.

7. Jennions MD, Petrie M. Variation in mate choice and mating preferences: a review of causes and consequences. Biol Rev Camb Phil Soc. 1997; 72:283–327.

8. Gabor CR, Halliday TR. Sequential mate choice by multiply mating smooth newts: females become more choosy. Behav Ecol. 1997; 8:162–166.

9. Bateman PW, Gilson LN, Ferguson JWH. 2001 Male size and sequential mate preference in the cricket *Gryllus bimaculatus*. Anim Behav. 2001; 61:631–637.

10. Houde AE. Mate choice based upon naturally occurring colour pattern variation in a guppy population. Evolution. 1987; 41:1–10.

11. Magurran AE. Evolutionary ecology: the Trinidadian guppy. Oxford: Oxford University Press; 2005.

12. Evans JP, Bisazza A, Pilastro A. Female mating preferences for colourful males in a population of guppies subject to high predation. J Fish Biol. 2004; 65:1154–1159.

13. Grether GF, Kolluru GR, Rodd FH, De la Cerda J, Shimazaki K. Carotenoid availability

affects the development of a colour-based mate preference and the sensory bias to which it is genetically linked. Proc R Soc Lond B Biol Sci. 2005; 272:2181–2188.

14. Karino K, Shimada Y, Kudo H, Sato A. Relative importance of the area and intensity of the orange spots of male guppies *Poecilia reticulata* as mating traits preferred by females. J Fish Biol. 2010; 77:299–307.
15. Becher SA, Magurran AE. Multiple mating and reproductive skew in Trinidadian guppies. Proc R Soc Lond B. 2004; 271:1009–1014.
16. Kelly CD, Godin JGJ, Wright JM. Geographical variation in multiple paternity within natural populations of the guppy (*Poecilia reticulata*). Proc R Soc Lond B. 1999; 266:2403–2408.
17. Pitcher TE, Neff BD, Rodd FH, Rowe L. Multiple mating and sequential mate choice in guppies: females trade up. Proc R Soc Lond B Biol Sci. 2003; 270:1623–1629.
18. Constanz J. Reproductive biology of the poeciliid fishes. In: Meffe GK, Snelson FF, Editors. Ecology and Evolution of Live Bearing Fishes (Poeciliidae), 1st ed. NY: Prentice Hall; 1989. p. 33–50.
19. Evans JP, Magurran AE. Multiple benefits of multiple mating in guppies. Proc Natl Acad

Sci. 2000; 97:10074–10076.

20. Evans JP, Gasparini C, Pilastro A. Female guppies shorten brood retention in response to predator cues. Behav Ecol Sociobiol. 2007; 61:719–727.

21. Karino K, Sato A. Male-biased sex ratios in offspring of attractive males in the guppy. Ethology. 2009; 115:682–690.

22. Karino K, Shinjo S. Female mate preference based on male orange spot patterns in the feral guppy *Poecilia reticulata* in Japan. Ichthyol res. 2004; 51:316–320.

23. Karino K, Shimada Y, Kudo H, Sato A. Relative importance of the area and intensity of the orange spots of male guppies *Poecilia reticulata* as mating traits preferred by females. J Fish Biol. 2010; 77: 299–307.

24. Haynes JL. Standardized classification of poeciliid development for life-history studies. Copeia. 1995; 1995:147–154.

25. Martyn U, Weigel D, Dreyer C. In vitro culture of embryos of the guppy, *Poecilia reticulata*. Dev dyn. 2006; 235:17–622.

26. Horton NJ, Lipsitz S. Review of software to fit generalised estimating equation regression models. Am Stat. 1999; 53:160–169.

27. Pilastro A, Evans JP, Sartorelli S, Bisazza A. Male phenotype predicts insemination success in guppies. Proc R Soc Lond B. 2002; 269:1325–1330.

28. Pilastro A, Simonato M, Bisazza A, Evans JP. Cryptic female preference for colorful males in guppies. Evolution. 2004; 58:665–669.

29. Janhunen M, Rudolfsen G, Kekalainen J, Figenschou L, Peuhkuri N, Kortet R. Spawning coloration and sperm quality in a large lake population of Arctic charr (Salmonidae: *Salvelinus alpinus* L.). Biol J Linn Soc. 2009; 98:794–802.

30. Peters A, Denk AG, Delhey K, Kempenaers B. Carotenoid-based bill colour as an indicator of immunocompetence and sperm performance in male mallards. J Evol Biol. 2004; 17:1111–1120.

31. Mehlis M, Hilke LK, Bakker TC. Attractive males have faster sperm in three-spined sticklebacks *Gasterosteus aculeatus*. Curr Zool. 2013; 59:761–768.

32. Locatello L, Rasotto MB, Evans JP, Pilastro A. Colourful male guppies produce faster and more viable sperm. J Evol Biol. 2006; 19:1595–1602.

33. Pitcher TE, Rodd FH, Rowe L. Sexual colouration and sperm traits in guppies. J Fish Biol. 2007; 70:165–177.

34. Skinner AMJ, Watt PJ. Phenotypic correlates of spermatozoon quality in the guppy *Poecilia reticulata*. Behav Ecol. 2007; 18:47–52.

35. Evans JP. Quantitative genetic evidence that males trade attractiveness for ejaculate quality in guppies. Proc R Soc Lond B. 2010; 277:3195–3201.

36. Eberhard WG, Cordero C. Sexual selection by cryptic female choice on male seminal products–a new bridge between sexual selection and reproductive physiology. Trend Ecol Evol. 1995; 10:493–496.

37. Simmons LW. Sperm competition and its evolutionary consequences in insects. Princeton, NJ: Princeton University Press; 2001.

38. Evans JP, Kelley JL, Bisazza A, Finazzo E, Pilastro A. Sire attractiveness influences offspring performance in guppies. Proc R Soc Lond B Biol Sci. 2004; 271:2035–2042.

39. Pilastro A, Gasparini C, Boschetto C, Evans JP. Colorful male guppies do not provide females with fecundity benefits. Behav Ecol. 2008; 19:374–381.

40. Moyano MPS, Silva P, Luppi T, Gavio MA. Female mate choice by chemical signals in a semi-terrestrial crab. J Sea Res. 2013; 85:300–307.

41. Barbosa F. An Integrative View of Postcopulatory Sexual Selection in a Soldier Fly:

Interplay Between Cryptic Mate Choice and Sperm Competition. In: Peretti A, Aisenberg A, Editors. Cryptic Female Choice in Arthropods. Netherlands: Springer; 2015. P. 385–401. https://doi.org/10.1007/978-3-319-17894-3_14

42. Dennenmoser S, Thiel M. Cryptic Female Choice in Crustaceans. In: Peretti A, Aisenberg A, Editors. Cryptic Female Choice in Arthropods. Netherlands: Springer; 2015. P. 203–237. https://doi.org/10.1007/978-3-319-17894-3_8

43. Thiel M, Hinojosa IA. Mating behavior of female rock shrimp *Rhynchocinetes typus* (Decapoda: Caridea)—indication for convenience polyandry and cryptic female choice. Behav Ecol Sociobiol 2003; 55:113–121. https://doi.org/10.1007/s00265-003-0677-1

44. Karino K, Utagawa T, Shinjo S. Heritability of the algal-foraging ability: an indirect benefit of female mate preference for males' carotenoid-based coloration in the guppy, *Poecilia reticulata*. Behav Ecol Sociobiol. 2005; 59:1–5.

45. Houde AE. Sex-linked heritability of a sexually selected character in a natural population of *Poecilia reticulata* (Pisces: Poeciliidae) (guppies). Heredity. 1992; 69:229–235. https://doi.org/10.1007/s00265-005-0001-3

46. Karino K, Haijima Y. Heritability of male secondary sexual traits in feral guppies in Japan.

J Ethol, 2001; 19:33–37.

47. Evans JP, Magurran AE Patterns of sperm precedence and predictors of paternity in the Trinidadian guppy. Proc R Soc B Biol Sci, 2001; 268:719–724. http://dx.doi.org/10.1098/rspb.2000.1577.

48. Neff BD, Wahl LM. Mechanisms of sperm competition: Testing the fair raffle. Evolution. 2004; 58:1846–1851.

49. Pilastro A, Mandelli M, Gasparini C, Dadda M, Bisazza A. Copulation duration, insemination efficiency and male attractiveness in guppies. Anim Behav. 2007; 74:321–328. http://dx.doi.org/10.1016/j.anbehav.2006.09.016.

Supporting information

S1 File. Raw data of experiments

Click here to access/download;Figure;Fig1.tiff

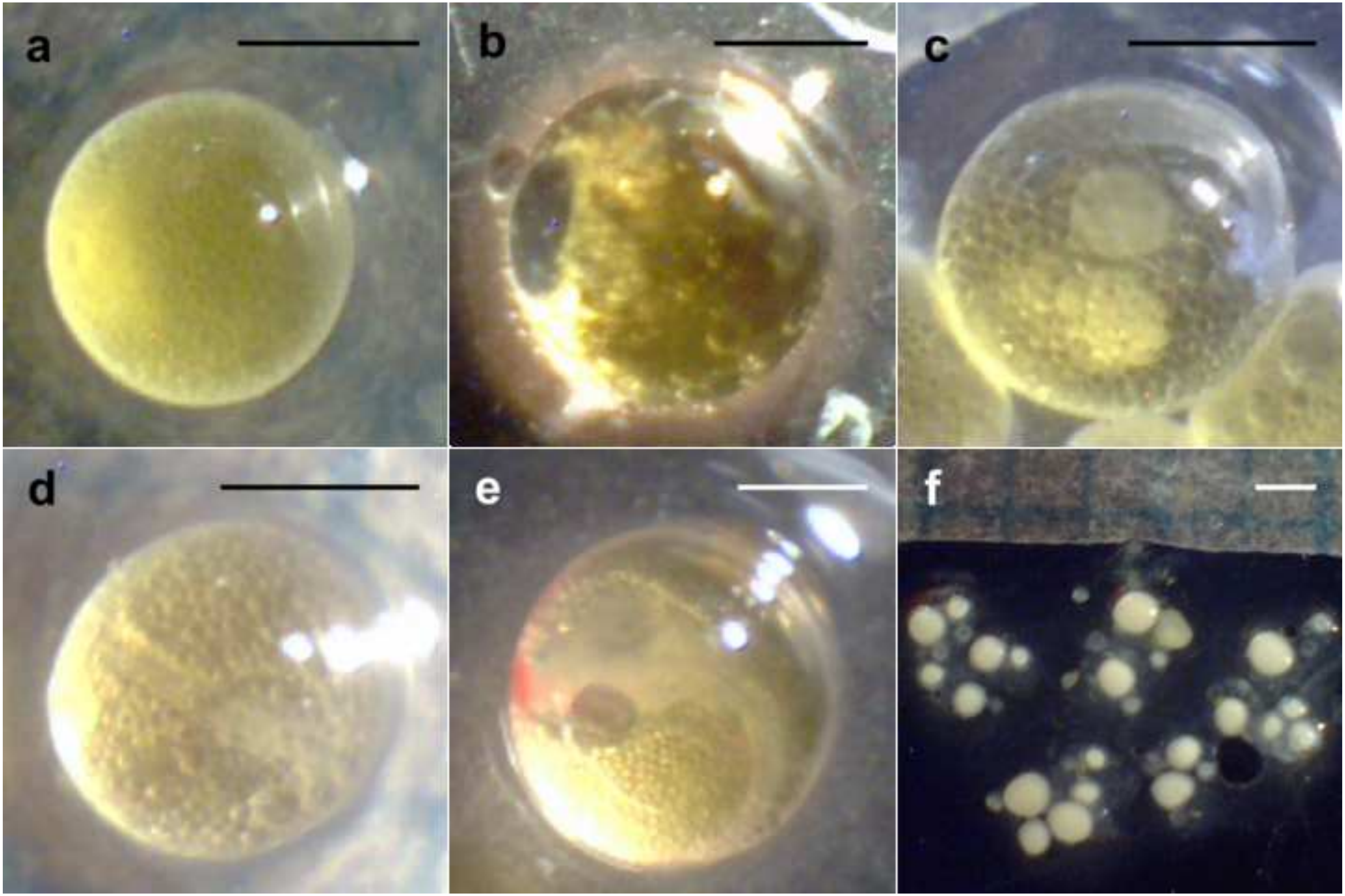

Figure 2

Click here to access/download;Figure;Fig2.tiff

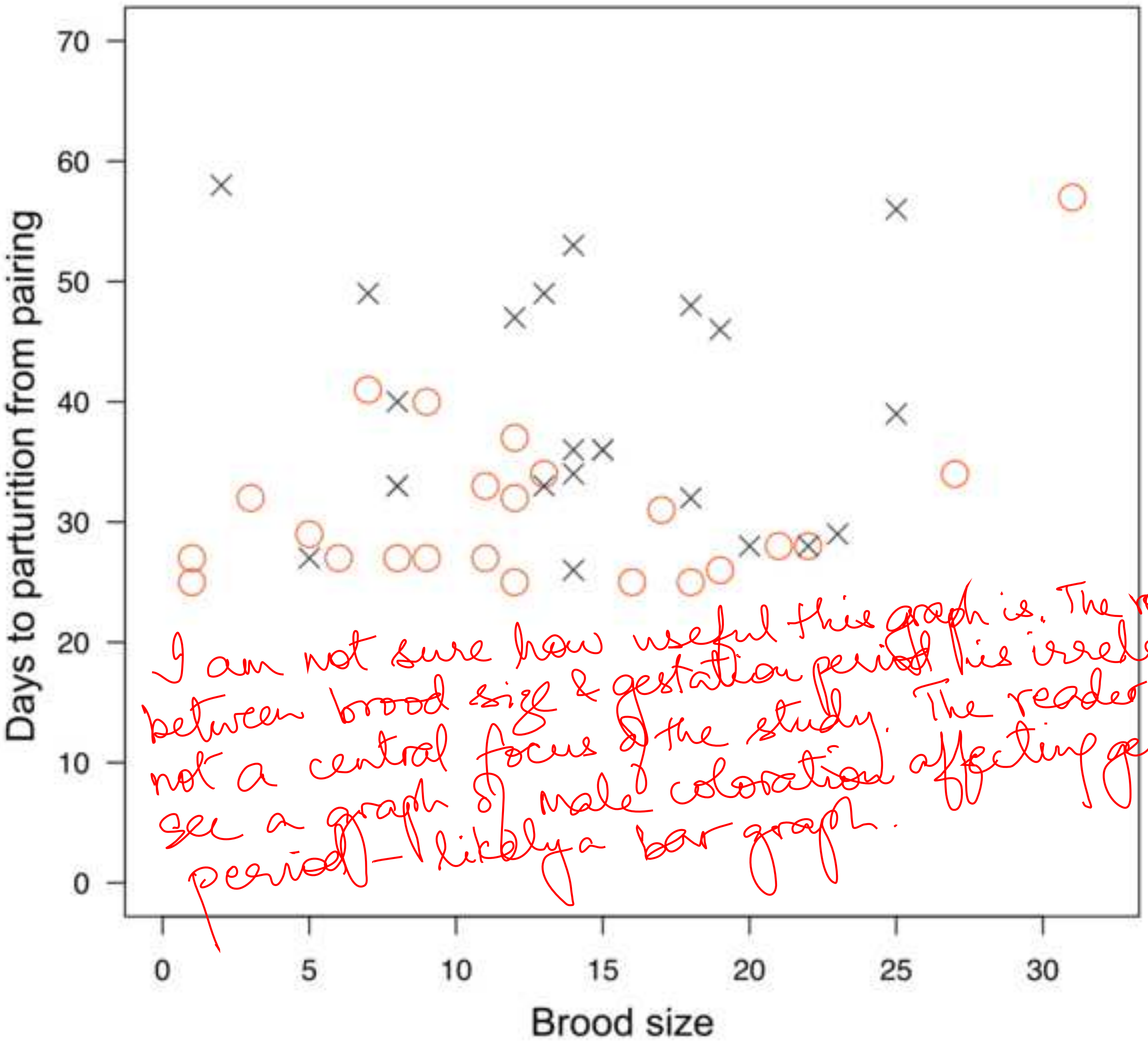

I am not sure how useful this graph is. The relationship between brood size & gestation period is irrelevant & not a central focus of the study. The reader needs to see a graph of male coloration affecting gestation period — likely a bar graph.

Figure 3

Click here to access/download;Figure;Fig3.tiff

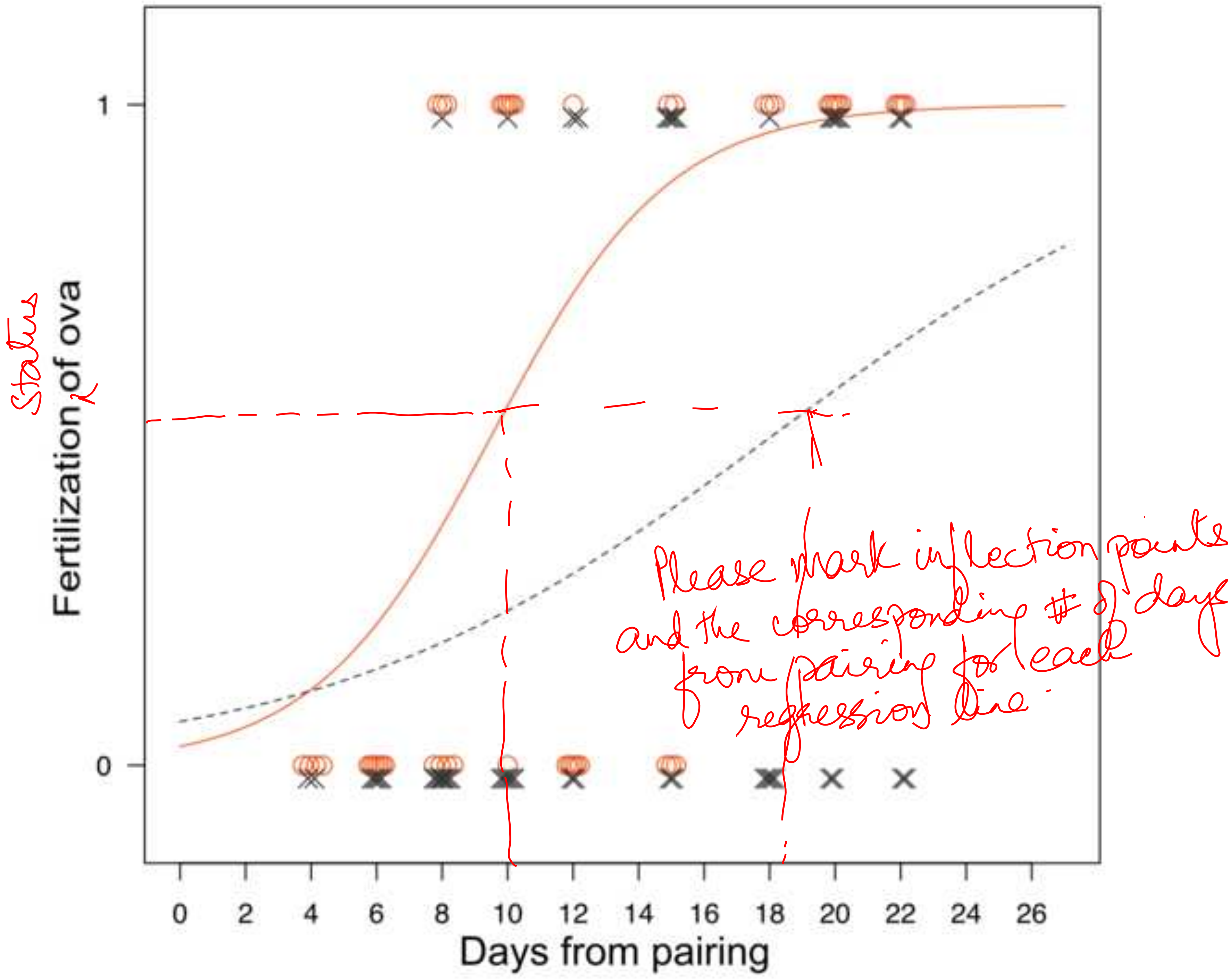

Click here to access/download
Supporting Information
supplemental file 1.xlsx
