# Supplementary material for: Male coloration affects female gestation period and timing of fertilization in the guppy (Poecilia reticulata)
Source: PLoS One. 2021 Dec 2;16(12):e0261004. doi: 10.1371/journal.pone.0261004 (PMC8639057; doi:10.1371/journal.pone.0261004)
Supplement: Attachment — Submitted filename: Response_to_Reviewers.docx [file pone.0261004.s003.docx]

Response to reviewers

We would like to express our appreciation for the reviewers’ suggestions for revising our manuscript. Addressing their comments has significantly improved the manuscript. The reviewers’ comments are appended hereafter in italics, and our responses are provided in regular font. The line numbers refer to those of the revised manuscript unless otherwise noted, in which the corresponding changes have been highlighted in the revised manuscript with changes tracked.

*Journal Requirements:*

*When submitting your revision, we need you to address these additional requirements.*

*1. Please ensure that your manuscript meets PLOS ONE's style requirements, including those for file naming. The PLOS ONE style templates can be found at*

*https://journals.plos.org/plosone/s/file?id=wjVg/PLOSOne_formatting_sample_main_body.pdf and*

*https://journals.plos.org/plosone/s/file?id=ba62/PLOSOne_formatting_sample_title_authors_affiliations.pdf*

>>We ensured that revised manuscript meets PLOS ONE’s style requirement.

*Reviewers' comments:*

*Reviewer's Responses to Questions*

*Comments to the Author*

*1. Is the manuscript technically sound, and do the data support the conclusions?*

*Reviewer #1: Yes*

*Reviewer #2: Yes*

*2. Has the statistical analysis been performed appropriately and rigorously?*

*Reviewer #1: No*

*Reviewer #2: Yes*

>>We have revised the statistical analysis according to the comments by reviewer #1. Details are described later.

*3. Have the authors made all data underlying the findings in their manuscript fully available?*

*Reviewer #1: Yes*

*Reviewer #2: Yes*

*4. Is the manuscript presented in an intelligible fashion and written in standard English?*

*Reviewer #1: No*

*Reviewer #2: Yes*

>> The revised manuscript has been edited by a professional language editing service (highlighted in gray). We have attached a Certificate of English Editing.

*5. Review Comments to the Author*

*Reviewer #1: This is an interesting and well-conceived study aimed at testing the prediction that female guppies delay egg maturation and fertilization when mated with a less colourful male.*

*The results seems to support this hypothesis, as the time between mating and parturition is longer in the female mated with drab males as compared to those mated with colourful males. Through dissections, the authors demonstrated that egg maturation proceeds faster in the colourful group.*

*I think that this may be a valuable contribution, but I have also a few suggestions that may be useful to revise the MS.*

*English requires extensive editing, and I suggest a revision from a native English speaker.*

>> We appreciate the comment. According to the comments by reviewer #1, the revised manuscript has been edited by a professional language editing service (highlighted in gray).

*A general comment: as you also reckon, it is difficult to distinguish between male-controlled and female-controlled effects of mating on fertilization. It may be that the quantity of ejaculate transferred during mating affects subsequent ovulation processes. Previous studies that used artificial insemination failed to find an effect of male colouration, but equal number of bundles were used. It may be interesting to test if different number of sperm inseminated affects gestation length.*

>>Thank you for the comment. Evans & Magurran (2000, [19] in the References list) showed that females that mated multiple males had a shorter gestation period than females that mated with a single male. From this comment of reviewer #1, we expect that the findings of Evans & Magurran (2000) can also be explained by the quantity of ejaculate transferred during mating. On the other hand, Pilastro et al. (2008) reported that adjusted ejaculate quantities did not affect the gestation period in artificial insemination. Therefore, we have added a discussion on the possible effects of the different quantities of sperm between colorful and drab males on the female gestation period (Lines 305-308, 315-316).

*Statistical methods:*

*1) It is not clear if gestation data were tested for normality. From a visual inspection it seems that a log transformation should be applied.*

>> Based on the result of the Kolmogorov-Smirnov test with Lilliefors significance correction, the gestation period data were not significantly normally distributed (D=0.175, df=46, p=0.001). Therefore, we log transformed the gestation period data (KS test in post-transform; D=0.130, df=46, p=0.050) and applied Box-Cox transformation (D=0.129, df=46, p=0.054). Because the log-transformed values fit the model better in the following test, we used log-transformed values for the analysis. We added the information that the gestation period was log-transformed (Lines 205-207).

*2) I do not see any good reason to use GEE. Data from tetrads in the parturition exp. are not repeated measures, rather they are not independent, and I think a generalized linear mixed model should be more appropriate. Male ID and tetrad can be entered as random factors, and male colour as fixed factor. Probably results will not change much.*

>> Thank you for your helpful suggestion. We have now used a generalized linear mixed model for the analysis of gestation data in the revised manuscript. We used the log-transformed gestation period as a dependent variable, the male coloration (colorful/drab) as a fixed factor, and the tetrad ID as a random factor. As suggested by reviewer #1, males were not used repeatedly in the parturition experiment. Therefore, we excluded male ID from the model. In addition, female SL and brood size were excluded from the model, according to the comment in the “other points” section of reviewer #1 and the comment by reviewer #2. The results did not change significantly. We have revised the description of the “statistical analyses” (Lines 202-205) and “results” (Lines 235-237, Table 1).

*3) The use of GEE may be more appropriate for the exp. on egg maturation, although here also a more commonly used generalized LMM may be used too. Indeed, it is not the same male that it is measured at different times, but different females mated with the same male (i.e. not independent) dissected once at different times after mating.*

>> We have used a generalized linear mixed model for analysis of the laparotomy experiment in the revised manuscript. We used tetrad and male ID as random factors. The results were the same as those when using GEE. We have accordingly revised the description of the “statistical analyses” (Lines 210, 212-214) and “results” (Table 2).

*Other points:*

*Table 1. You should check if male type remains significant after removing for non-significant covariates (brood size and female size).*

>> We removed the brood size and female size from the model. The effect of male type remained significant. We have revised the results (Lines 235-237) and Table 1 accordingly.

*Line 225: please also give the mean difference +/- SD between that two males.*

>>We have added the mean difference+/- SD between that two males: “the difference in the orange area between colorful and drab males pairs was 7.9 % ± 5.0 %” (Lines 228-229).

*Line 315: there is a study demonstrating a first male fertilization success when female influenced is controlled using artificial insemination (Magris et al. 2017 Animal Behaviour 131:45-55).*

>> Thank you for your helpful suggestion. We have cited this reference and revised the sentence (Lines 328-332).

*Line 318: this is not exact. Your results demonstrate that there is a delay in fertilization over days. Experiments on trade-up (e.g. Evans et al. 2001, Pitcher et al. 2003) involved subsequent matings occurring over a much shorter time (1h up to 24 h). While the observed delay in ovulation mat increase the temporal window within which female cryptic choice can occur, your data do not directly demonstrate that this is involved in trading up. So your conclusion is that delayed fertilization increase the opportunity for cryptic female choice and trading up, not that it is involved in trading up, which is most probably determined by females controlling the number of sperm transferred during matings.*

>> Thank you for your helpful suggestions. We agree with this comment. We have revised this conclusion accordingly (Lines 334-335).

*Line 328: I like this argument, this is an interesting point.*

>> Thank you. We also look forward to future research.

*Reviewer #2: Dear Authors,*

*This is a well designed study, and the findings are useful to the scientific community. I have one major change that I would like to see in Figure 2. All edits and comments are embedded within the attached PDF file of your manuscript.*

*Best wishes!*

>> Thank you for the comments. We appreciate correcting the English. The language corrected according to the comments by reviewer #2 is highlighted in blue in the revised manuscript with changes tracked. We have revised the manuscript as follows in response to other comments.

**Lines100-102（previous manuscript）, Line 99-100（revised manuscript）**

We received the following comments from the reviewer #2 to our sentence (“In this study, we investigated whether the sexual attractiveness of mated male would affect gestation period and timing of fertilization in female guppies.”)

: *“didn’t Evans & Magurran alredady study this?”*

>> We have responded as follows:

Evans & Magurran (2000, [19] in the References list) showed that females that mated with multiple males had a shorter gestation period than females that mated with a single male. And it was suggested that females that mated with single male may have delayed the process of fertilization. In this study, we directly tested whether male sexual attractiveness affected the timing of fertilization when a females mated with a single male, through laparotomy experiments.

**Line118-122（previous manuscript）**

Reviewer #2 commented on the paragraph of *“Experimental design” as “Move to introduction after prediction statement”*.

We have moved that paragraph to lines 101-105.

**L131-132（previous manuscript）**

Reviewer #2 commented on the total and standard length as *”caudal fin included or not ?”.*

We have added that the total length included the caudal fin and that standard length did not include the caudal fin (Lines 129-130).

**L136（previous manuscript）**

Reviewer #2 commented on the light for photography as *a ”type of bulb? Color temperature?”*.

We have added the information that we used the krypton bulb of daylight (Sunlamp KR R45, Asahi). (Lines 135-136)

**L140（previous manuscript）**

Reviewer #2 commented regarding the male photography as *“Did you ensure that caudal fin is fully opened to avoid errors in area measurement?”*.

We have added the following to the section on male coloration measurement:

“The male body was fully immersed in the anesthetic solution, and after complete lack of movement, the male body was gently shaken in the solution to open the caudal fin. We took some photographs and selected the clearer photographs, and the ones in which the caudal fin was fully opened for analysis (Lines 137-140)”

**L147（previous manuscript）**

Reviewer #2 commented on the procedure of visually contacting the test females with many males prior to pairing, as to whether all females were placed in a small tank or each female was placed in a small tank.

We have added that the two females in a tetrad were placed in a small tank for visual contact between the females and the males (Line 150).

**Line 204（previous manuscript）**

Reviewer #2 commented on the analysis of parturition experiment as:

The brood size and the female standard length can be taken out of the model if these effects were not significant.

We changed the method of analysis from general estimating equations (GEE) to generalized linear mixed models (GLMM) according to the comment of reviewer #1. Moreover, the effects of brood size and female size were not significant. Therefore, we removed them from the final model (Lines 235-237, Table 1).

**Line210-211（previous manuscript）**

Reviewer #2 commented on the analysis of parturition experiment that tetrad and male ID can be taken out of the model if these effects were not significant.

We have also changed this analysis from GEE to GLMM according to the comment of reviewer #1. Because we used tetrad and male ID as random factors in the GLMM, these factors were used to detect the relationship between the fixed variables and the dependent variable, but did not detect a direct effect on the dependent variable. Therefore, we left them in the model as random variables.

**Table 1**

According to the changes in the results of analyses, we revised Table 1. In addition, we revised Fig 1 according to the comment from reviewer #2.

**Line 250 （previous manuscript）**

Reviewer #2 commented on the result that excluded some females from the analysis, as “*Number of females remaining in laparotomy treatment was?*”

We have added number of females remaining in the analysis of laparotomy treatment:

“Finally, the numbers used in the analysis were 46 females that paired with colorful males and 46 females that paired with drab males” (Lines 257-258).

**Fig. 2**

Reviewer #2 commented that Fig.2 should change to likely a bar graph. Based on this comment, we have revised Fig. 2. Because original data of the gestation period were not normality, we have changed Fig.2 to boxplots.

**Fig.3**

Reviewer #2 commented on Fig.2 as “*Please make inflection points and the corresponding No of days from pairing for each regression line.*”.

We have accordingly revised Fig. 3.
