# Supplementary material for: Male coloration affects female gestation period and timing of fertilization in the guppy (Poecilia reticulata)
Source: PLoS One. 2021 Dec 2;16(12):e0261004. doi: 10.1371/journal.pone.0261004 (PMC8639057; doi:10.1371/journal.pone.0261004)
Supplement: Attachment — Submitted filename: Response to Reviewers.docx [file pone.0261004.s004.docx]

Response to reviewers

We would like to express our appreciation for the reviewers’ suggestions for revising our manuscript. We have improved the manuscript according their comments. The reviewers’ comments are appended hereafter in italics, and our responses are provided in regular font. The line numbers refer to those of the revised manuscript, in which the corresponding changes have been highlighted in the revised manuscript with changes tracked.

*Journal Requirements:*

*Please review your reference list to ensure that it is complete and correct. If you have cited papers that have been retracted, please include the rationale for doing so in the manuscript text, or remove these references and replace them with relevant current references. Any changes to the reference list should be mentioned in the rebuttal letter that accompanies your revised manuscript. If you need to cite a retracted article, indicate the article’s retracted status in the References list and also include a citation and full reference for the retraction notice.*

>> We have reviewed our reference list and ensured that it is complete and correct. We have not cited papers that have been retracted.

*Additional Editor Comments (if provided):
The experimental design is sound and valid. Statistical analyses were appropriate, after the authors' first revision. I invite the authors to address some minor concerns raised by the reviewers to further improve the quality of the manuscript.*

>> Thank you for your comments. We have revised our manuscript as follows:

*Reviewers' comments:*

*Reviewer's Responses to Questions*

***Comments to the Author***

*1. If the authors have adequately addressed your comments raised in a previous round of review and you feel that this manuscript is now acceptable for publication, you may indicate that here to bypass the “Comments to the Author” section, enter your conflict of interest statement in the “Confidential to Editor” section, and submit your "Accept" recommendation.*

*Reviewer #3: (No Response)*

*Reviewer #4: (No Response)*

*2. Is the manuscript technically sound, and do the data support the conclusions?
The manuscript must describe a technically sound piece of scientific research with data that supports the conclusions. Experiments must have been conducted rigorously, with appropriate controls, replication, and sample sizes. The conclusions must be drawn appropriately based on the data presented.*

*Reviewer #3: Yes*

*Reviewer #4: Yes*

*3. Has the statistical analysis been performed appropriately and rigorously?*

*Reviewer #3: Yes*

*Reviewer #4: Yes*

*5. Is the manuscript presented in an intelligible fashion and written in standard English?*

*PLOS ONE does not copyedit accepted manuscripts, so the language in submitted articles must be clear, correct, and unambiguous. Any typographical or grammatical errors should be corrected at revision, so please note any specific errors here.*

*Reviewer #3: No*

*Reviewer #4: No*

>> The revised manuscript has been edited by a professional language editing service. We have attached a certificate for English editing.

*6. Review Comments to the Author*

*Reviewer #3: Guppies are a model organism for sexual selection, and there are many empirical studies on mate choice, but in fact most of them are on pre-copulation mate choice, and empirical research has been limited on post-copulation mate choice (cryptic female sperm choice), although its existence has been predicted. The present study is a good study that reported results supporting the existence of cryptic female sperm choice in guppies through a simple problem formulation and carefully manipulated experiments. In addition, Materials and Methods have been properly improved in revised version.
I think this MS is suitable for publication in PLoS ONE.*

*I have only a few minor comments.*

>> Thank you for your comments. We have responded to the comments as follows:

*Comments to the authors*

*1) Lines 33-35 in revised MS*

*As reviewer1 mentioned, your results demonstrate that there is a delay in fertilization over days, but your data do not directly showed female cryptic choice.*

*Therefore, you should revise here as you revised in Line 333-335 in revised MS..*

>>Thank you for pointing this out. We revised the conclusion of the abstract as follows:

“Our findings show that differences in the timing of fertilization according to attractiveness of the mate increase the opportunity for cryptic female choice and trading up.” (Lines 36-37)

*2) Lines 99-100 in revised MS*

*According to your MS, it seems that the effects of sexual attractiveness of mated males on gestation period in guppies have already revealed in Karino and Sato 2009 (Lines 95-96 [21]).*

*Did you confirm the previous results, or what are the new points for investigation of gestation period in the present study?*

>> In this study, we aimed to determine whether “the difference in gestation period according to male attractiveness in a parturition treatment experiment” and “the difference in the timing of fertilization according to male attractiveness in a laparotomy treatment experiment” correspond. We were afraid that the gestation period or timing of fertilization could be affected by female size or water temperature. In addition, in Karino and Sato (2009), females mated with males only once, but in this study, females were placed in the same tank as males for 24 h. Therefore, we wanted to measure the gestation period and timing of fertilization under the same conditions.

In addition, control data were needed to support the possibility that unfertilized eggs in the parturition treatment were female manipulation rather than failure in pregnancy.

Based on the above, we examined the female gestation periods in this study. In the revised manuscript, we have changed the end of the introduction as follows:

“In this study, we directly tested whether the sexual attractiveness of mated males affects the timing of fertilization in female guppies. We observed whether the ova were fertilized by opening their abdomens between 2 and 22 d after pairing with colorful or drab males. Additionally, we measured the gestation period (duration from mating to parturition) between females that mated with colorful males and those that mated with drab males, and compared whether the difference in the timing of fertilization according to male coloration reflected the difference in the gestation period.” (Lines 102-108）

*3) Line 99 in revised MS*

*I think "In this study, we directly tested whether ..." would be more appropriate as you answered to reviewer 2.*

>>We revised this sentence according to this comment as follows:

“In this study, we directly tested whether the sexual attractiveness of mated males affects the timing of fertilization in female guppies.” (Lines 102-103)

*4) Lines 281-283 in revised MS*

*I agree with your idea. However, you don't explain whether other possibilities remain or not. Therefore, if other possibilities remain, I recommend you to revise the sentence as follows:*

*These results suggest that the difference in gestation periods possibly caused by the difference in timing of fertilization is affected by mate ornamentation.*

>> We agree with your comment. We have revised this sentence according to this comment and revision from a professional language editing service. “These results suggest that the difference in gestation periods depending on mate ornamentation is possibly caused by the difference in timing of fertilization.” (Lines 284-285)

*5) Lines 287-288 in revised MS*

*I think the following expression would be more appropriate.*

*"because there are possibilities that some females that paired with drab males failed to fertilize their ova. "*

>> We have revised this sentence according to this comment and revision from a professional language editing service. “because it is possible that some females that paired with drab males failed to fertilize their ova.” (Lines 289-290)

*Reviewer #4: This is a informative experiment to reveal fundamental reproduction behaviour of guppy in selecting preference mate, which is important to under how female select potential mate and controlling sperm insemination to produce quality offspring. Authors also provided interesting hypothesizes in the introduction section. Overall, MM, Results and Discussion sections support the hypothesizes and addressing objectives of the study. However, conclusion part was not highlighted the aim of the study and hypothesis of the study. Suggest author to summarize the hypothesis of the study at conclusion section. In addition, discussion section (Line 322-330), how female guppy control preference male sperm for fertilisation? this is interesting point to highlight in the study. Suggest authors discuss a little further about this point.*

*language need to be improved, some sentences are difficult to follow especially in the MM & discussion sections.*

>> Thank you for your helpful comments. We have put a section “Conclusions” to which we have added a summary of the hypothesis of the study (Lines 355-365). In addition, we added to the discussion section a report from a previous study on how females bias paternity toward the second male when they mate sequentially with two males (Lines 329-336).

To improve the language of our manuscript, we have received the service of a professional language editing service and have revised our manuscript.
